# Considerations for adaptive design in pediatric clinical trials: study protocol for a systematic review, mixed-methods study, and integrated knowledge translation plan

**DOI:** 10.1186/s13063-018-2934-7

**Published:** 2018-10-19

**Authors:** Lauren E Kelly, Michele P Dyson, Nancy J Butcher, Robert Balshaw, Alex John London, Christine J Neilson, Anne Junker, Salaheddin M Mahmud, S Michelle Driedger, Xikui Wang

**Affiliations:** 1 Department of Pediatrics and Child Health, The University of Manitoba, 405 Chown, 753 McDermot Ave., Winnipeg, MB R3E0T6 Canada; 2 Clinical Trials Platform, George & Fay Yee Centre for Healthcare Innovation, Winnipeg, MB Canada; 3 Department of Community Health Sciences, University of Manitoba, Winnipeg, MB Canada; 4 Department of Pediatrics, University of Alberta, Edmonton, AB Canada; 5 Child Health Evaluative Sciences, The Hospital for Sick Children Research Institute, Toronto, ON Canada; 6 Data Science Platform, George & Fay Yee Centre for Healthcare Innovation, Winnipeg, MB Canada; 7 Center for Ethics and Policy, Carnegie Mellon University, Pittsburgh, PA USA; 8 Neil John Maclean Health Sciences Library, University of Manitoba, Winnipeg, MB Canada; 9 Department of Allergy and Immunology, British Columbia Children’s Hospital, Vancouver, BC Canada; 10 Vaccine and Drug Evaluation Centre, University of Manitoba, Winnipeg, MB Canada; 11 Department of Statistics, University of Manitoba, Winnipeg, MB Canada

**Keywords:** Adaptive design, Clinical trials, Child health, Extrapolation, Research ethics

## Abstract

**Background:**

Although children have historically been excluded from clinical trials (CTs), many require medicines tested and approved in CTs, forcing health care providers to treat their pediatric patients based on extrapolated data. Unfortunately, traditional randomized CTs can be slow and resource-intensive, and they often require multi-center collaboration. However, an adaptive design (AD) framework for CTs could be used to increase the efficiency of pediatric CTs by incorporating prospectively planned modifications to CT methods without undermining the integrity or validity of the study. There are many possible adaptations, but each will have ethical, logistical, and statistical implications. It remains unclear which adaptations (or combinations thereof) will lead to real-world improvements in pediatric CT efficiency. This study will identify, evaluate, and synthesize the various regulatory, ethical, logistical, and statistical considerations and emerging issues of AD in CTs that could be used to evaluate the use of drugs in children.

**Methods/design:**

Following the development of a peer-reviewed search strategy, a systematic review on AD in CTs will be conducted. Data on regulatory, ethical, logistic, and statistical considerations as well as population and trial design characteristics will be synthesized. A mixed-methods study including surveys and focus groups with regulators, research ethics board members, biostatisticians, clinicians, and scientists, as well as representatives from patient groups and the public will evaluate the opportunities and challenges in applying AD in trials enrolling children and propose recommendations on best practices.

**Discussion:**

This study will deliver practical recommendations on the use of AD in pediatric CTs. Collaboration and consultation with national and global partners will ensure that our results meet the needs of researchers, regulators, and patients, both locally and globally, and that they remain current and relevant by engaging a wide variety of stakeholders. Overall, this research will enrich the knowledge base regarding if, how, and when AD can be used to answer research questions with fewer resources while still meeting the highest ethical standards and regulatory requirements for CTs. In turn, this will result in increased high-quality clinical research needed by health care providers so they have access to appropriate, population-specific evidence regarding the safe and effective use of medicines in children.

## Background

Evidence-based pharmacotherapy includes the conscientious use of the best available knowledge alongside patient values and clinical expertise to guide decision making at the bedside. According to a 2014 report by the Council of Canadian Academies, at least 50% of infants, children, and youth are prescribed at least one medicine each year [1]. Unfortunately, pediatric safety and effectiveness data are often not available for the medicines used, as clinical trials (CTs) have historically been conducted in adults. Without high-quality evidence from CTs, health care providers are forced to select and dose medicines in children based on evidence established in other populations (i.e., adults, older children, or other conditions for which the drug has been tested). Compared with adult populations, drug response in children is known to be more heterogeneous [2–5]. Children may respond differently to medicines resulting from non-linear growth in drug metabolism enzymes [2], and they often experience unique conditions (e.g. necrotizing enterocolitis, juvenile idiopathic arthritis, Kawasaki disease, Duchenne muscular dystrophy, leukemia) with unique disease etiologies compared with the adult population. The use of medicines without appropriate pediatric evidence increases variability in prescribing and can lead to over- or under-dosing; this places children at an increased risk for adverse events as well as efficacy or treatment failure. The lack of data on pediatric drug response means that approximately two-thirds of children in outpatient clinics receive an off-label (no authorized indication for this age group) medicine [6], and this increases to almost 90% in neonatal intensive care units [1, 7]. While extrapolation of effectiveness evidence obtained from adult populations may be acceptable under certain conditions, conducting more CTs with children would undoubtedly ensure that health care providers have better quality, age-appropriate evidence on the safety and effectiveness of drugs used to treat children.

Ethical and scientific reasons for the historical lack of CTs with children have been well described [1]. To overcome the challenge of having a relatively small population from which to recruit trial participants, combined with the limitations of traditional CTs, there is an urgent need for CT designs that present feasible alternatives while meeting ethical, regulatory, and methodological standards. Traditional CT design leads to protocols with rigid operating characteristics, such as total sample size, probabilities associated with fixed randomization ratios, number and composition of treatment groups, etc. In all prospective CTs, key study design elements such as the primary outcome, clinically meaningful treatment differences, and expected response variability are prespecified [8]. The success of CTs thus critically depends on the accuracy of key assumptions made prior to the recruitment of the first patient [9]. However, particularly in pediatric trials, there will often be a very limited pool of data upon which to base these essential assumptions and design decisions. Importantly, no use is made of the accumulating information through the trial, except for interim analyses to monitor safety, and effectiveness observations are analyzed only after all participants have been treated.

The principles of adaptive designs (ADs) present an alternative to traditional randomized CTs intended to increase ethical conduct and study efficiency. An adaptive design clinical trial (ADCT) is defined by the Food and Drug Administration (FDA) as “a study that includes a prospectively planned opportunity for modification of one or more specified aspects of the study design and hypotheses based on analysis of data (usually interim data) from subjects in the study” [10]. One of the defining features of an ADCT is that the possibility of adaptations is preplanned and prespecified [10], allowing for modifications to trial design characteristics in light of information accumulated during the CT. These adaptations can come in many forms, including (but not limited to) modifications to trial eligibility criteria, treatment regimens and dosing, study sample size re-estimation to enhance power based on accumulating information, the statistical analysis plan, the choice of the primary outcome, outcome adaptive randomization to assign more participants to the potentially superior treatment, or stopping of the trial if interim analysis reveals a low probability of detecting a difference between treatment and control (futility) [11, 12]. Multiple names are used for certain ADCTs, e.g., basket trials, platform trials, umbrella trials, drop the loser, play the winner, seamless phase II/III, and others [11]. ADCTs have been used at all stages of the clinical development process and can reflect either Bayesian or frequentist approaches to trial design or analysis. Adaptations are predominantly made based on data acquired from study participants, but they can also arise from external unexpected information (e.g., data from a separate pharmacokinetic study become available) [10].

Adaptive designs typically have lower expected sample sizes or lower realized sample sizes compared with fixed designs, for the same statistical power (e.g., by reducing sample sizes up to 60% compared to “fixed” sample sizes) [13]. However, their maximal sample size is typically larger or can yield improved information on how an intervention should be applied (e.g., to which subgroup, or at which dose) [9, 14]. ADCTs may also identify ineffective interventions earlier than traditional CTs, thus reducing overall subject burden (i.e., the number of children required to be involved in a study) and trial costs [8]. The judicious and widespread use of approved medicines for children requires evaluation beyond what CTs require for marketing authorization. Regulators in Europe have suggested that, particularly where extrapolation cannot be used with certainty, there may be a role for ADCTs early in the drug development pathway, but that discussions should be held with regulators early in the protocol development process [15]. A report published in 2006 indicated that regulatory experience with ADCTs was limited [16]. Furthermore, the opinions of health technology assessment bodies that determine which medications will be covered (or on formulary) for particular conditions based on evidence from ADCTs have not been evaluated.

The principles of AD were introduced in the 1950s [17] and have been applied previously in some pediatric CTs with some success. In fact, an analysis of 24 pediatric CTs conducted between 1963 and 2005 with sequential designs showed a significant reduction in sample size relative to what would have been calculated with a traditional fixed-sample design [13]. More recently, there has been discussion among European Union (EU) regulatory bodies with respect to the validity of extrapolation from adults to children [18]. Within this discussion, it has been recommended that during drug development pediatric investigation plans be adaptive and integrated into the development plans for adults [15].

The PREMILOC trial in particular provides a strong example of the potential benefits of AD [19]. The results of this study identified a significant increase in survival without bronchopulmonary dysplasia in preterm babies given low-dose hydrocortisone administered within the first 10 postnatal days compared with placebo. The researchers performed sequential interim analyses estimating parameters of efficacy and safety to avoid recruiting additional participants once either efficacy or futility had been established [19]. This adaptive approach allowed researchers to stop the trial as soon as a clear signal of efficacy had been demonstrated, eliminating the need to randomize potentially hundreds of babies to receive placebo had the trial continued until a traditionally determined sample size was obtained.

While ADCTs appear to be advantageous to child health researchers, the scientific (including statistical), regulatory, ethical, and logistical considerations for ADCTs in children have yet to be established. Currently, it is not known when, how, and what combinations of adaptations work best to answer which research questions. Previous work by Hatfield et al., who evaluated the characteristics of AD approaches used in registered CTs between 2000 and 2014, reported variability in the type and combinations of adaptations applied in CTs involving all populations [20]. However, this study did not extract data on age groups from included trials. CT registries, ethics boards, and regulators report seeing an increase in the use of AD [20], but barriers to implementing ADCTs exist. Kairalla et al. formally reviewed barriers to ADCTs in 2012 [8]. Barriers to the uptake of ADCT not specific to child health include a lack of knowledge and expertise among researchers, few case studies, and a lack of infrastructure support for the planning of ADCTs [8]. Moreover, statistical analysis methods for AD are unconventional and not widely understood, and although potentially desirable compared with traditional designs, AD itself is not free of ethical issues. These barriers create uncertainty and hesitation among researchers and funding agencies as to how study methods and findings will be perceived by the community. Concerns regarding the introduction of bias and the control of type I and type II error rates have also contributed to hesitation among research funders and peer reviewers [8]. Furthermore, these frequentist concerns may also outweigh a more evidence-driven, Bayesian calculus of sample size, resulting in unnecessarily large samples and increased recruitment efforts. As education and expertise are reported barriers, developing and disseminating evidence tailored to the needs of various stakeholders on ADCT considerations is essential to familiarize researchers, ethics boards, regulators, journal editors, and families on the appropriate use of AD in CTs globally.

Following principles of integrated knowledge translation (IKT) [21], we have partnered researchers with knowledge user organizations (e.g., various Canadian CT networks, including those with as well as without a specific pediatric focus, and with CT networks in the USA and Europe) for this study. Principles of IKT mean that researchers and knowledge users/partners are involved in all stages of the research to ensure scientific, practical, and policy relevance. In this study, these knowledge users, researchers, and partner organizations have been involved in defining the research plan and will aid in interpreting the findings and disseminating the deliverables. The objective of this study is to identify, evaluate, synthesize, and disseminate regulatory, ethical, logistical, and statistical facilitators and challenges of AD application in pediatric CTs. These data will inform the development of educational materials regarding if and how AD can be used to increase efficiency in answering clinical research questions. Moreover, in true IKT fashion, some of our collaborating partners are study authors.

## Methods/design

This study will address previously identified barriers to the adoption of AD in child health CTs (incorrect use, lack of knowledge, few cases, lack of support, ethical challenges, regulatory evaluation, methodological and statistical issues) [8] through a systematic review and mixed-methods study. To increase appropriate use of AD in CTs, this study will include a comprehensive IKT component.

### Systematic review

The systematic review has been designed in accordance with the Preferred Reporting Items for Systematic Review and Meta-Analysis Protocols (PRISMA P) [22]. Specifically, the review will evaluate where, when, and how AD has been used in CTs in children and adults. Findings in pediatric and adult trials will be compared to investigate potential differences in application of AD in pediatric and adult populations.

#### Eligibility criteria

Published CT protocols, reports, and a secondary analysis using an adaptive method will be included. ADCTs will be defined based on the FDA description provided earlier [10], allowing also for formal incorporation of relevant external information that becomes available. Interventions will be limited to drugs and vaccines, as there are separate regulatory guidance and ethical considerations for device and behavioral studies in Canada and elsewhere. CTs of all diseases and populations (children and adults) will be included, as long as they involve at least one pharmacological agent. CT trial registrations (e.g., ClinicalTrials.gov) will not be included at this stage, as these registries rarely provide any useful information on methods or justification of methods that would identify a trial as an ADCT. The time of publication will be restricted from 2010 to 2018, for feasibility purposes. Also, because FDA guidance [10] on ADCTs was first issued in 2010, we expect to find more rigorous and consistent ADCT application after this time.

#### Search strategy

An experienced research librarian (CJN) will design a preliminary search strategy for ADCTs using Ovid MEDLINE. Search results will be limited to English, French, and Dutch (the languages in which our project team is fluent) and articles published between 01January 2010 and the 2018 search date. No other search filters will be applied. The search strategy will be peer-reviewed by a second, independent librarian according to Peer Review of Electronic Search Strategies (PRESS) guidelines [23], and the finalized search strategy will be translated for use in additional bibliographic databases (see the Appendix).

#### Sources and selection process

The literature search will incorporate the following bibliographic databases: MEDLINE (Ovid), Embase (Ovid), CENTRAL (Wiley), International Pharmaceutical Abstracts (Ovid), and MathSciNet. All titles and abstracts will be evaluated by two independent reviewers for eligibility. Full-text review, conducted in duplicate, will determine the nature of any relevant adaptations made in each CT. Any studies where it is unclear if an adaptation was made will be reviewed by the senior author and study team. Furthermore, references from recent reviews on AD from 2016 forward will be reviewed for inclusion.

#### Data management and collection

EndNote will be used to manage abstract screening and function as part of the study database. Data will be extracted using standardized forms in REDCap [24] following the appropriate training and approvals by the University of Manitoba. The data to be extracted will include characteristics of the CT population (age group, disease, location of recruitment), study objective, key characteristics of the CT design and statistical analysis plan (nature of the intervention(s), use of any of the control groups’ primary and secondary outcomes, planned sample size and sample size methodology, intended analyses and how closely they reflect the study design) type of adaptation(s), name of applied AD method, rationale for adaptation, reported challenges, or study limitations categorized as regulatory, ethical, logistical, statistical, or other. Data extraction with REDCap will also allow us to capture random distributions of sample sizes, conditional on individual study assumptions. Data collection procedures will include the use of detailed screening logs for recording details of studies warranting exclusion. If data are available from both a protocol and a report for the same study, the methods applied will be compared. Modifications and the justification for the ADCT modifications will be sought from corresponding study authors (if not reported). The work of two individual data extractors will be assessed for agreement.

#### Risk of bias

The methodological quality of individual studies will be evaluated using the Cochrane risk of bias tool [25]. The domains of Cochrane’s tool for assessing risk of bias are selection bias (random sequence generation and allocation concealment), performance bias (blinding of participants and personnel), detection bias (blinding of outcome assessment), attrition bias (incomplete outcome data for each of the main outcomes), reporting bias (selective reporting), and other sources of bias including the identification, attribution, and resolution of adverse events.

#### Data synthesis and analysis

The strength of the body of evidence will not be evaluated, as the analysis will be primarily descriptive, and there will be no pooling of results in a meta-analysis. Subgroup analyses will include CTs with children (only participants younger than 18 years), mixed populations of both children/adults, and only adults. The rationale for evaluating AD trials with adults and children separately is to compare the type of adaptation and frequency of AD use between the two groups. A separate analysis comparing registration/protocol/publication discrepancies for the same study will be conducted to evaluate what modifications were made and if there were unanticipated challenges before the study was conducted. A potential exploratory subgroup analysis is planned for neonatal studies (participants younger than 28 days at the time of recruitment). Results from this review will inform the instruments developed for the second stage of this research: the mixed-methods study.

### Mixed-methods study

The objective of the mixed-methods study is to elicit input from a broader set of interested stakeholders to evaluate the importance and scope of regulatory, ethical, logistical, and statistical issues related to ADCTs involving children. Key priorities related to ADCTs in children will be distinguished from AD facilitators and challenges relevant in all populations. As the review may be unable to identify regulatory challenges, the mixed-methods study will obtain practical feedback from a regulatory and health technology assessment perspective. We will elicit this input using both an online survey and a 2-day face-to-face meeting. Approval for this study will be obtained prospectively from the University of Manitoba Health Research Ethics Board.

### Online survey

The aim of the online survey is to have participants evaluate the importance of regulatory, ethical, logistical, and statistical features of ADCT, identify additional potential challenges not identified in the review, and classify which aspects are specific in a pediatric context relative to AD in general (all populations).

#### Target study population and recruitment

We have partnered with several CT networks that will help recruit our mixed-methods study population. We aim to recruit participants with a broad set of expertise, understanding, and familiarity with CTs as well as a knowledge of the challenges involved in applying AD in trials enrolling children. Specifically, our partner CT networks will use their membership lists to recruit the following types of participant groups: regulators, health technology assessors, research ethics board members, biostatisticians, clinicians, researchers, and members of an interested public (i.e., recruited from patient/parent/family groups). We will also invite authors of included studies from the systematic review. To ensure a broad representation, we will use social media and other strategies to recruit additional participants with experience in pediatric CTs.

Our intent is to recruit 100 participants from these different strategies, with an aim to have maximum variation purposive sampling [26]. Namely, we will seek to recruit 15–20 members from each participant group category identified above. We recognize that recruitment for some participant group categories will be more difficult to achieve; that is, recruiting regulators or health technology assessors may be harder than recruiting biostatisticians, clinicians, and researchers. Thus, we will seek to recruit at least 10 people who identify with each category. The stakeholder type will be self-selected, acknowledging that many individuals may represent multiple groups. Individuals who represent multiple stakeholder categories will be placed in a group with fewer participants. This redistribution will take place at the end of recruitment, according to patterns observed during the recruitment period. We will attempt to extend survey invitations until at least 10 individuals in each category have participated, or until a period of 2 months has passed.

The online survey will ask specific questions regarding the importance (using a numerical scale) of regulatory, ethical, logistical, and statistical considerations identified in the systematic review and will also solicit any additional concerns relating to various adaptations in CTs involving children. Participants will also be asked if each consideration is pediatric-specific or would be generally applicable in all CTs. At the end of the survey, participants will be asked if they are interested in attending a 2-day facilitated meeting involving focus group discussions. We would seek to recruit approximately 24 participants from the survey sample to participate in the 2-day facilitated meeting. Recognizing that there may be challenges in recruiting equal numbers of members for each participant group, we would continue recruiting until we had a good representation for each participant group type. As we aim to recruit a representative sample within our broad survey participants, each survey will be assigned a numeric identifier in order to facilitate a combined analysis of input from the online survey and the 2-day facilitated meeting.

### Two-day facilitated meeting

The aim of the facilitated meeting is to arrive at a stronger rationale focused on priorities, preferences, implications, acceptability, and consequences surrounding AD in pediatric CTs. We will also pilot and seek feedback on four developed case examples to be used for teaching important concepts related to ADCTs. Specifically, using a combination of strategies — small group discussions, opportunities to assess group cohesion around certain concepts using “group voting” technology — the 2-day meeting will assess areas of convergence around priority elements and possible barriers to the use of ADCT and will present new ideas the research team may not have identified in previous project stages [27].

#### Two-day meeting process

One week prior to the meeting, participants will receive a summary report of findings from the systematic review and a descriptive summary of the survey results. The first day of the meeting will consist of a discussion of the systematic review and survey results through a combination of facilitated group discussions to assess within- and between-group perspectives. Discussion groups will be separated by stakeholder type in the morning, and randomly assigned mixed stakeholder groups (a maximum of eight people) will meet in the afternoon to assess how participant perspectives change when exposed to the priorities and opinions of other stakeholder group types. At the end of the first day, the project team will also assess priority elements to bring forward for discussions on the second day. Using clickers, participants will present anonymized responses to questions surrounding the priorities, preferences, implications, acceptability, and consequences of AD presented in the cases. The goal on the second day will be to elicit feedback on appropriate AD application in child health research using four distinct case examples. Case examples based on the literature review and survey findings will be created by the project team to further elicit preferences and considerations regarding the application of AD and will be presented in one of the educational video deliverables. Educational materials will be made available online and open access (see the section on knowledge translation).

#### Analysis

Small and large group discussions from over the 2 days will be recorded after obtaining written consent from participants, transcribed verbatim, audio-verified, and analyzed using NVivo 11™ [28]. Transcripts will be coded using a constant comparative and concept development approach [29, 30]. Two team members will develop a coding framework of descriptive surface content by independently reviewing transcripts and developing draft coding domains with definitions and refining through consensus. We will develop coding domains inductively from the data, and qualitative content will be sorted along the five prespecified consideration domains: regulatory, ethical, logistical, statistical, and other. By focusing on descriptive surface content, research assistants will be trained to systematically apply the coding guide to the dataset (after achieving inter-rater reliability scores of 0.85 or higher) [31]. We will compare and contrast early attitudes from the survey responses to participant discussions over the 2-day meeting. This will enable a more fulsome understanding of participant attitudes and an assessment of how these attitudes may have shifted (or not). Results from the qualitative study will be reported following conventional guidelines [32, 33].

#### Ensuring data quality

Standard metrics of reliability and validity do not apply in the same way when blending quantitative and qualitative data in mixed-methods designs. To ensure interpretation validity of study findings, we will involve our diverse knowledge users and partner organizations to assist in the analysis. Moreover, as we will be tapping into different stakeholder participant groups, their engagement in the study by completing the survey and/or participating in the 2-day face-to-face meeting will provide a broader perspective to contextualize the analysis. Study participants who agree will be sent a draft report of the mixed-methods results (aggregate results so as to maintain confidentiality) and requested to comment on the overall credibility of our interpretation as well as on any concerns they may have regarding major issues or errors. These data will be included in the final analysis. Data on the application of AD in pediatric CTs will be triangulated in two ways. First, the findings from the systematic review will inform the scope of regulatory, ethical, logistical, and statistical issues to be assessed through the surveys, facilitated group discussions over the course of the 2-day meeting, researcher reflections, and participant feedback as described above to ensure confirmability of findings [31, 34]. Second, the different stakeholder types involved in participant groups for the mixed-methods portion of the research, when combined with the interdisciplinary backgrounds of the full research team and knowledge user partners, will ensure that different perspectives inform the analysis.

### Knowledge translation

All study materials will be made freely accessible through publication in open access journals and disseminated through the chimb.ca website as well as national and international conferences. We will develop educational materials, tailored to the needs of our stakeholders, which will be shared through social media, websites, newsletters, and meetings. Stakeholders for this project include clinicians, academic researchers (including methodologists and statisticians), ethicists, ethics board members, regulators, journal editors, and members of the public. We will ensure meaningful engagement with the public through collaboration with the provincial Strategy for Patient-Oriented Research (SPOR) support unit platforms. Through a diverse group of partnerships, including the Pediatric Trials Network (USA), the Pediatric Clinical Research Infrastructure Network (Europe), KidsCAN Trials (Canada), and Clinical Trials Ontario, we will have access to a wide audience in both Canada and abroad. To ensure the ongoing relevance of our results, we will also work with these partners to obtain feedback on the utility and function of our educational materials through the administration of usability surveys. The goal of our partnerships is to harmonize methodological approaches and increase the efficiency of pediatric CTs globally. Biannual teleconferences will be set to discuss ongoing AD trial challenges and methodological barriers and to share training materials. This opportunity will also be used to discuss and address regulatory hurdles in various jurisdictions globally.

An educational portal will be created and will be openly available on the chimb.ca website to share the case examples, webinars, and training videos. We will use the knowledge gained through the systematic review and mixed-methods study to create online webinars relating to specific adaptations and create a downloadable training manual supported by short videos. Each of these components will be tailored for specific stakeholder audiences based on the mixed-methods study. Metrics related to web traffic will be collected as a proxy of the uptake of the educational materials. By breaking topics into small digestible pieces, we will create opportunities for the community to utilize the portal to answer specific questions or for in-depth training. By creating an annotated bibliography of all CTs using AD identified from our systematic review, we will promote acceptance and familiarity with ADCTs. We are committed to providing ongoing support for AD through the Centre for Healthcare Innovation, which houses experts in CT methods and biostatistics. Support will be offered to researchers through free 1-h consultations, followed by a fee for service or opportunity for collaboration model.

## Discussion

Within the realm of pediatric CTs, it is critical to facilitate efficient multi-center CTs in order for studies to be adequately powered to evaluate drug safety and effectiveness. The findings of this project will determine if and how AD can answer research questions more efficiently in pediatric populations to facilitate multi-centre trials and minimize the burden that such research places on patients and their families. The support of national training infrastructure and methodological expertise will be necessary to encourage researchers to apply AD. The commonly multi-centre nature of CTs in child health warrants agreement between regulators and research ethics boards of the suitability and challenges of innovative CT design. We recognize and acknowledge that agreement does not automatically imply that the correct conclusions have been reached, and we will use this possibility as an opportunity to create materials and interventions for education. Our project team will engage with regulators in Canada, the USA, and Europe to evaluate their perceptions and training needs for reviewing CTs using AD. If ADCTs can indeed be shown to make better use of scarce funding and CT resources, they will most certainly allow more research questions to be answered with more high-quality evidence. More relevant and reliable evidence will mean that health care providers will have access to the appropriate, population-specific evidence they require to ensure the safe and effective use of medicines in children.

Based on previous studies [8, 35] and discussions within the research community, we know there is a knowledge and training gap on the use of AD in CTs. Appropriate use and successful uptake of AD will require changes to perceptions of the clinical research community and updated policies — changes that must be based on evidence obtained systematically from a variety of stakeholders. Paramount is the need to identify when and how AD can increase CT efficiency. By creating new and by synthesizing existing knowledge, this study will lead to increased confidence and familiarity with AD in CTs for the research community and the public. This may, in turn, increase the likelihood that ADCTs will receive funding, since reviewers can then be directed to evidence on the strengths and weaknesses of ADCT. We aim to change the thought process and support how CTs are designed; this will have an impact extending far beyond child health research.

Potential limitations of the systematic review include the impact of publication bias. As positive and novel findings are more likely to be published, this may skew the analysis of included studies. While large-scale efforts to reduce publication bias (e.g., negative results journals, open reviewing) are beyond the scope of this project, we will endeavor to objectively critique the literature and report these limitations in resulting deliverables. Furthermore, planned studies that may have encountered the most significant barriers to implementing an AD design for a CT may not have begun at all (e.g., planned trials unable to overcome ethical or regulatory hurdles for the proposed adaptations); these would not be reflected in the published literature. Secondly, there are various views on the ethical considerations for CTs involving children [36, 37]. It is feasible that disagreements in ethical and scientific principles for involving children in research could be encountered during our focus groups. We will present all arguments provided in our deliverables to provide a clear picture of all opinions presented. Through involvement of a diverse group of stakeholders, including the public, we expect and encourage alternative views on the application of innovative trial methods to maximize the breadth and scope of our education materials. Our research will be useful only to the extent that the community can access it. Ensuring that our deliverables reach those planning CTs will also be a challenge. To this end, we have engaged a diverse group of national and global partners to help facilitate dissemination, and we plan to meet bimonthly with our partners to ensure that our deliverables meet their needs and the needs of their networks. Finally, it is inevitable that there will be some identified barriers (e.g., funding models that do not account for flexible sample sizes) for which we may be unable to propose solutions. These will be noted and will set the agenda for future projects.

## Appendix

### Finalized literature search strategy for ADCTs

Search syntax guide:

**Table:**

• / indicates a MeSH subject heading search	
• .ti,ab,kf,kw. Indicates a keyword search of the fields: title, abstract, author keyword word, author keyword	
• adjn indicates proximity, where n = allowable distance between keyword terms	
• * indicates truncation	

**Table:**

Ovid MEDLINE(R) Epub Ahead of Print, In-Process & Other Non-Indexed Citations, Ovid MEDLINE(R) Daily, and Ovid MEDLINE(R) < 1946 to Present>	
**#**	**Searches**
1	Adaptive Clinical Trials as Topic/
2	Adaptive Clinical Trial/
3	(Adapt* adj3 (design* or rule* or randomiz* or randomis* or trial*)).ti,ab,kf,kw.
4	“Flexible design”.ti,ab,kf,kw.
5	“Group sequential”.ti,ab,kf,kw.
6	(Bayesian adj3 (design or adapt* or model* or approach)).ti,ab,kf,kw.
7	((data or result or results) adj3 (Continu* adj3 (reasses* or assess*))).ti,ab,kf,kw.
8	(Sample adj3 (reestimat* or “re estimate*” or increase* or adjust* or modify or modified or modification*)).ti,ab,kf,kw.
9	(“stopping rule*” or “stopping boundar*”).ti,ab,kf,kw.
10	(“drop the loser” or “pick the winner” or “play the winner”).ti,ab,kf,kw.
11	(“O’Brien” adj2 Fleming).ti,ab,kf,kw.
12	(Repower* or “re power*”).ti,ab,kf,kw.
13	(“Dose selection” adj2 rule*).ti,ab,kf,kw.
14	((“Phase 2-3” or “phase II-III” or “Phase 2/3” or “phase II/III” or “Phase 2b-3” or “phase IIb-III” or “Phase 2b/3” or “phase IIb/III”) and seamless).ti,ab,kf,kw.
15	“alpha spending”.ti,ab,kf,kw.
16	or/1-15
17	limit 16 to yr = “2010-2017”
18	limit 17 to (dutch or english or french)

## Abbreviations

AD: adaptive design
ADCT: adaptive design clinical trial
CT: clinical trails
FDA: Food & Drug Administraion
IKT: integrated knowledge translation
